# *Giardia duodenalis* and *Cryptosporidium* spp. in meat purchased at retail in Canada

**DOI:** 10.1016/j.fawpar.2026.e00357

**Published:** 2026-08-26

**Authors:** Harriet Merks, Jessica Remedios, Nicol Janecko, Brent R. Dixon

**Affiliations:** a Bureau of Microbial Hazards, Food and Nutrition Directorate, Health Canada, Ottawa, ON K1A 0K9, Canada; b Quadram Institute Bioscience, Norwich NR4 7UQ, United Kingdom; c Foodborne Disease and Antimicrobial Resistance Surveillance Division, Public Health Agency of Canada, Guelph, ON N1H 7M7, Canada

**Keywords:** *Giardia*, *Cryptosporidium*, Parasites, Retail meat, Canada

## Abstract

The enteric protozoan parasites, *Giardia duodenalis* and *Cryptosporidium* spp., or their DNA, have been reported in surveillance studies on fresh produce and in shellfish worldwide, but have been reported only rarely in meat or on the carcasses of meat animals. The objective of the present study, therefore, was to determine the prevalence of *G. duodenalis* and *Cryptosporidium* spp. in retail meat purchased in three Canadian provinces as part of FoodNet Canada's (Public Health Agency of Canada) retail surveillance activities. Packages containing chicken, beef, pork or veal were purchased at grocery stores in FoodNet Canada sentinel sites. Following DNA extraction from the meat samples, nested-PCR and DNA sequencing were used to detect parasite-specific sequences. *Giardia duodenalis* DNA was detected in 1.5% of 468 meat packages, while *Cryptosporidium* spp. DNA was present in 2.1% of 468 packages. Parasite DNA was detected in meat purchased in all three Canadian provinces sampled, with no apparent seasonal variation in prevalence. The presence of *G. duodenalis* and *Cryptosporidium* spp. DNA in retail meats was unexpected, but does not necessarily confirm the presence of viable parasites. It does, however, suggest possible cross-contamination either at the abattoir or during processing. Considering that the meats tested in this study are very often cooked before consumption, the risk of meat-borne transmission of these parasites is likely minimal.

## Highlights

• *Giardia duodenalis* DNA was detected in 1.5% of Canadian retail meat packages.
• *Cryptosporidium* spp. DNA was detected in 2.1% of Canadian retail meat packages.
• Findings suggest possible cross-contamination at the abattoir or during processing.
• The risk of meat-borne transmission of these parasites is considered to be minimal.

## 1 Introduction

The enteric protozoan parasites *Giardia duodenalis* and *Cryptosporidium* spp. are commonly reported in humans and a wide range of domestic animals and wildlife worldwide. Both are important causative agents of gastrointestinal diseases, namely giardiasis and cryptosporidiosis, respectively. Transmission of infectious *G. duodenalis* cysts or *Cryptosporidium* spp. oocysts occurs either directly through contact with fecal material from an infected human or animal, or indirectly through contaminated water or foods (Dixon, 2021; Zahedi and Ryan, 2020).

Cysts of *G. duodenalis* and oocysts of *Cryptosporidium* spp., or their DNA, have been detected worldwide during surveillance studies of a wide variety of foods (Dixon, 2016; Ryan et al., 2019). Numerous foodborne outbreaks of cryptosporidiosis have been reported worldwide, particularly associated with fresh produce, unpasteurized milk and apple cider (Robertson and Chalmers, 2013; Ryan et al., 2018, Ryan et al., 2019; Zahedi and Ryan, 2020). Most foodborne outbreaks of giardiasis have occurred in the United States and, while the type of food involved in these outbreaks was often not determined, fresh produce was most commonly implicated, along with a variety of processed foods and unpasteurized milk (Dixon, 2021; Ryan et al., 2019).

*Giardia duodenalis* and *Cryptosporidium* spp. are both very commonly reported to infect domestic animals and wildlife (Feng and Xiao, 2011; Ryan et al., 2021) but, while unpasteurized milk has been implicated in a number of illness outbreaks, meat and organ tissues have only very rarely been reported to be contaminated with *G. duodenalis* or *Cryptosporidium* spp., or associated with foodborne outbreaks.

A possible example of a giardiasis outbreak of animal origin occurred in Turkey following the consumption of tripe soup (Karabiber and Aktas, 1991). The tripe was prepared from sheep, and the authors suggested that the animal may have been infected and that cysts could have remained viable in intestinal crevices during cooking. In Japan, a small outbreak of acute gastroenteritis due to cryptosporidiosis was reported in 2006 (Yoshida et al., 2007). The suspected sources of this foodborne outbreak included a raw meat dish called “Yukke” (Korean-style beef tartar) and raw liver. In both of these outbreaks, the association with meat consumption was only circumstantial and full investigations into the source of infection may not have been carried out.

A few studies have confirmed the presence of *G. duodenalis* and *Cryptosporidium* spp. in the feces of meat animals at slaughter. For example, Kaneta and Nakai (1998) reported the presence of *C. muris* oocysts in 4.7% of adult cattle in a slaughterhouse in Japan. Koyama et al. (2005) reported a 1.5% prevalence of *C. andersoni* in adult cattle in a slaughterhouse in Japan. A study in Ireland reported the presence of *C. parvum* oocysts in the feces of 7.3% of cattle presented for slaughter (Duffy et al., 2003; Moriarty et al., 2005). More recently, Ma et al. (2019) investigated the occurrence of *Cryptosporidium* and *Giardia* in slaughterhouse water in China by PCR and sequencing, and reported 0% and 11.1% detection in the samples, respectively.

Little information is available, however, regarding the presence of *Giardia* cysts or *Cryptosporidium* oocysts, or their DNA, on either the carcasses of meat animals or on cuts of meat (Robertson, 2014). A study in Ireland specifically sampled areas of beef carcasses (rump tissue and brisket tissue), which had previously been shown to become contaminated with feces during slaughter, but did not detect *Cryptosporidium* on any of the carcasses (Duffy et al., 2003; Moriarty et al., 2005). Rai et al. (2008) reported a high PCR prevalence of *Giardia* and *Entamoeba* in raw goat meat in India, and a lower prevalence of *Cryptosporidium*. Robertson and Huang (2012) analyzed cured meat products for the presence of *Cryptosporidium* oocysts due to possible contamination at the manufacturer during a large waterborne outbreak in northern Sweden in 2010. The authors reported the detection of a single putative oocyst by immunofluorescence microscopy which could not be confirmed due to its deformation. In a preliminary study conducted in our laboratory, we detected both *G. duodenalis* and *Cryptosporidium* spp. in raw meats purchased at retail. Chicken breasts, minced beef and pork chops were all positive by nested PCR, with some samples confirmed positive by immunofluorescence microscopy (Dixon, 2009). A recent study in Ghana investigated the presence of multiple bacterial and parasitic pathogens in raw beef samples, but *Cryptosporidium* was not detected (Adjei et al., 2022).

In light of the scarcity of information on the potential contamination of meats with *G. duodenalis* and *Cryptosporidium* spp., the objective of the present study was to determine the PCR prevalence of these important enteric parasites in retail meats from three Canadian provinces. This information will be important in determining any potential risk of foodborne transmission from retail meats. This work was performed as part of a targeted study by FoodNet Canada led by the Public Health Agency of Canada (PHAC).

## 2 Materials and methods

### 2.1 Retail sampling

Packages of chicken breasts, ground beef, ground pork, and veal (assorted cuts) were purchased by FoodNet Canada (PHAC) samplers at grocery stores in three Canadian provinces. Retail sentinel sites included: British Columbia (Abbotsford, Burnaby and Chilliwack), Alberta (Calgary and Central zone) and Ontario (London), representing over 5% of the Canadian population. The purchased meat was pre-packaged, and all information provided on the package label and the retail store was recorded. All of the retail meat purchased for this study was derived from Canadian producers, none was imported. Sampling occurred weekly in 2015 for chicken, beef, and pork, and in 2017 for veal. A total of 468 packages of meat were purchased, including 94 packages of chicken breasts, 93 packages of ground beef, 94 packages of ground pork, and 187 packages of veal (assorted cuts). All meat samples were purchased fresh and unfrozen.

Immediately after purchase, chicken, beef, and pork packages were placed in coolers lined with freezer packs, and shipped via overnight courier to the Health Canada laboratory in Ottawa, ON for testing. These meat samples were originally tested in our laboratory for the presence of *Toxoplasma gondii* DNA (Iqbal et al., 2018) and were subsequently frozen at −20 °C until DNA extraction and PCR testing for *G. duodenalis* and *Cryptosporidium* spp. could be performed for the present study. Veal samples were purchased and immediately shipped to the FoodNet laboratory in Guelph, ON for microbiological testing before being frozen at −20 °C and shipped by overnight courier to the Health Canada laboratory where they were stored until further processing and parasite testing could be completed.

### 2.2 Sample processing

Upon receipt at the Health Canada laboratory, packages of meat were inspected for any leaks or damage, and package information was logged. Samples were thawed and, using sterile knives, 25 g of finely chopped chicken, 25 g of ground beef and ground pork, and 20 g of finely chopped veal, were weighed directly into Ziploc® bags. The remainder of each package was stored at −20 °C.

A 25 g sample of ground beef was prepared as a positive process control for each testing batch. This sample was a composite prepared by combining small aliquots from multiple packages of ground beef. The composite was artificially spiked with 50 μL suspensions of *G. duodenalis* cysts and *C. parvum* oocysts (Waterborne Inc., New Orleans, LA), representing approximately 62,500 cysts and oocysts, respectively. The artificially spiked composite was then stored at 4 °C overnight and processed the following day alongside test samples. The purpose of this positive process control was to confirm that all steps in the meat testing procedure (i.e., elution and concentration, lysis, and extraction) were working effectively.

### 2.3 Elution and concentration of *Giardia* cysts and *Cryptosporidium* oocysts

Two hundred mL of phosphate buffered saline containing 0.01% Tween20 (PBST), pH 7.4, was added to each Ziploc® bag containing meat samples. Bags were sealed and placed on an orbital shaker for 10 min at 120 rpm. Immediately after shaking, all contents of the bag were poured into a modified bottle-top vacuum filter unit, which included a 55 μm filter. The filtrate was then transferred to four 50 mL conical-bottomed tubes and centrifuged at 2000 ×*g* for 15 min at 4 °C. Supernatant was aspirated and discarded, leaving approximately 5 mL, and the pellet, undisturbed. Pellets were resuspended and combined into a single 50 mL tube. Phosphate buffered saline (PBS) was used to rinse the tubes, and the rinse was combined with the pooled pellets and centrifuged at 2000 ×*g* for 15 min at 4 °C. Supernatant was discarded to within 0.5 mL of the pellet and the tube was vortexed. One hundred μL of concentrated suspension was then used for lysis as described below. The remaining volume of the concentrate was stored at 4 °C until microscopy could be performed.

### 2.4 Lysis of *Giardia* cysts and *Cryptosporidium* oocysts

Lysis of concentrates was performed by repeated freeze/thaw of 100 μL concentrate suspended in 300 μL NucliSens EasyMag lysis buffer (bioMérieux, France). An additional sample was prepared as a positive lysis control and consisted of EasyMag buffer spiked with 50 μL suspensions of *Giardia* and *Cryptosporidium*, equivalent to approximately 62,500 cysts and oocysts, respectively. The purpose of this control was to confirm that the lysis procedure was effective in breaking open cysts and oocysts for the subsequent DNA extraction step. In addition, a negative lysis control was run with each batch of samples. This was a no-template control consisting of lysis buffer alone. The purpose of this negative lysis control was to ensure that no contamination occurred during this step in the analysis. Samples and controls were subjected to five alternating 2 min cycles of freezing in liquid nitrogen followed by thawing in a 95 °C water bath. Lysed samples were cooled to room temperature, after which 20 μL of 20 mg/mL Proteinase K solution (Qiagen, Hilden, Germany) was added, followed by overnight incubation at 56 °C in an Eppendorf Thermomixer R set to 450 rpm (Eppendorf, Hamburg, Germany).

### 2.5 DNA extraction

DNA extraction was performed using a NucliSENS EasyMag robotic extractor (bioMerieux, France) following manufacturer's instructions. The entire lysed sample, containing 100 μL of concentrate, 300 μL lysis buffer, and 20 μL proteinase K, was loaded onto the NucliSENS EasyMag robotic extractor. All samples were eluted in a final volume of 100 μL. Extracted DNA was generally used immediately as the template for PCR amplifications, although, in a few cases, it was necessary to store the DNA at −20 °C until PCR could be performed.

### 2.6 PCR amplification

A fragment of the *Giardia* 18S-rRNA ribosomal unit was amplified using the outer PCR primers Gia2029 and Gia2150c, followed by a second amplification using the nested PCR primers RH11 and RH4, generating 497 bp and 292 bp fragments respectively, as previously described (Appelbee et al., 2003) (Supplementary Table 1). The master mix and PCR conditions used in these amplifications were as per Merks et al. (2023).

To identify the presence of *Cryptosporidium*, a 769 bp fragment of the *Cryptosporidium* Outer Wall Protein (COWP) gene was amplified using the outer primers BCOWP F and BCOWP R (Pedraza-Díaz et al., 2001), followed by a second amplification using the nested PCR primers COWP cry-15 F and COWP cry-9 R (Spano et al., 1997), resulting in a 553 bp gene fragment (Supplementary Table 1). The master mix and PCR conditions used in these amplifications were as per Merks et al. (2023).

Both positive and negative PCR controls were added to each PCR run. Respectively, these controls were used to confirm that the PCR master mix and amplification conditions used were effective in amplifying the parasite DNA, and to confirm that the master mix had no contamination or non-specific PCR products. The positive PCR control included *Giardia* and *Cryptosporidium* DNA from a batch of previously prepared and evaluated control material. UltraPure™ RNase/DNase-free distilled water was used as a no-template negative PCR control.

A positive PCR result was based on the visualization of bands of the correct size following agarose gel electrophoresis of the nested PCR products. Amplicons of the correct size, as determined by agarose gel electrophoresis, were purified using either the QIAquick PCR purification kit or the QIAquick Gel Extraction kit (Qiagen, Mississauga, ON) following manufacturer's instructions.

In order to minimize any possibility of laboratory contamination of the meat samples or the concentrates, a high level of rigor was used throughout the preparation, processing and amplification of samples and controls, including the use of separate rooms for specific tasks, sterile hoods and dedicated thermocyclers.

The limit of detection of the *Giardia* 18S and *Cryptosporidium* COWP PCR assays described above was determined prior to the analyses of the retail meat samples. Specifically, an aliquot of beef concentrate was spiked with 50 μL each of *Giardia* cysts and *Cryptosporidium* oocysts (Waterborne Inc., New Orleans, LA), equivalent to 62,500 cysts and 62,500 oocysts. The spiked beef was then lysed and extracted as per the methods describe above. The final extracted volume of 100 μL DNA was then used to prepare a 1:10 serial dilution (i.e., undiluted, 1:10, 1:100, 1:1000 and 1:10,000) which was amplified by *Giardia* 18S and *Cryptosporidium* COWP PCR, and visualized by gel electrophoresis to determine the limits of detection. As a control, 50 g aliquots of unspiked beef, chicken and pork were washed, concentrated, lysed, and extracted using the same methods to determine if the meat matrices themselves produced any non-specific background signal with these genes.

### 2.7 DNA sequencing

The purified PCR products were subjected to bi-directional, cycle sequencing using a BigDye Terminator v3.1 Cycle Sequencing Kit (Applied Biosystems, Waltham, MA) following manufacturer's instructions. Amplified sequence products were purified using a Wizard MagneSil Sequencing Reaction Clean-up System (Promega, Madison, WI), and capillary electrophoresis was performed on an ABI 3500 Genetic Analyser (Applied Biosystems, Waltham, MA). DNA sequences were assembled, edited and aligned using SeqScape v3 software (Applied Biosystems, Waltham, MA). Nucleotide BLAST analysis (Altschul et al., 1990) was performed on the assembled bidirectional consensus sequences using megablast with the GenBank standard nucleotide collection database.

### 2.8 Microscopy

Microscopical examination was performed on all sequence-confirmed PCR-positive samples. For *Giardia* and *Cryptosporidium*, a 100 μL sample of concentrate was stained with 20 μL of fluorescein isothiocyanate (FITC)-labeled monoclonal antibody preparation, Crypto/Giardia Cel Reagent (Cellabs Pty. Ltd., Brookvale, New South Wales, Australia). Parasite suspensions were incubated for 1 h at room temperature followed by a Dulbecco's phosphate buffered saline (DPBS) (Invitrogen Corp., Carlsbad, CA) wash and centrifugation at 10,000 ×*g* for 10 min. The supernatant was removed, leaving a total of 100 μL, including the pellet, undisturbed.

Twenty μL of the stained sample suspension was pipetted onto a microscope slide; a coverslip was applied, and the slides were examined on a Nikon Eclipse 80i epifluorescence microscope (Nikon Canada, Inc., Mississauga, ON). A blue filter block (excitation, 450–490 nm; emission, 505 nm) was used to visualize FITC-monoclonal antibody-stained *Giardia* cysts and *Cryptosporidium* oocysts. Up to a maximum of five slides were examined for each PCR-positive sample.

## 3 Results

*Giardia duodenalis* and *Cryptosporidium* spp. DNA was detected in retail meat samples collected in all three Canadian provinces. *Giardia duodenalis* DNA was detected in 7 (1.5%) of the samples of retail meat tested, including chicken breasts, ground beef and veal (Table 1). None of the ground pork samples were positive for *G. duodenalis*. *Cryptosporidium* spp. DNA was detected in 10 (2.1%) of the meat samples, with positive PCR results from all meat types. *Cryptosporidium* spp. DNA was significantly more likely to be found in ground beef than in the other meats tested (OR = 10; *p* < 0.01; CI 2.56–39.83) (Supplementary Table 2). One veal sample was found to contain the DNA of both parasites.

**Table 1:** Occurrence of *Giardia duodenalis* and *Cryptosporidium* spp. DNA in retail meats in Canada.

Retail meat	No. packages tested^1^	*Giardia duodenalis* PCR-positive (%)	*Cryptosporidium* spp. PCR-positive (%)
Chicken breasts	94	2 (2.1)	1 (1.1)
Ground beef	93	2 (2.2)	7 (7.5)
Ground pork	94	0	1 (1.1)
Veal (assorted cuts)	187	3 (1.6)	1 (0.5)^2^
Total	468	7 (1.5)	10 (2.1)

1 Only one chicken breast per package was tested.

2 One sample was PCR-positive for both *C. parvum* and *G. duodenalis.*

Process controls, consisting of ground beef spiked with *G. duodenalis* cysts and *C. parvum* oocysts, were PCR-positive in all sample batches, indicating that all steps in the meat testing procedure (i.e., elution and concentration, lysis, and extraction) were working effectively.

The limit of detection determinations for the PCR assays used in this study demonstrated that *Giardia* DNA could be detected down to a dilution factor of at least 1:10,000, while *Cryptosporidium* DNA could be detected down to a dilution factor of 1:1000. Based on the initial spike level of 62,500 *Giardia* cysts and *Cryptosporidium* oocysts, this corresponds to detection limits of approximately 6.25 cysts and 62.5 oocysts. None of the unspiked meat samples produced any non-specific background signal following PCR amplification of the *Giardia* 18S gene nor the *Cryptosporidium* COWP gene.

Neither *G. duodenalis* cysts nor *Cryptosporidium* spp. oocysts were observed in any of the retail meat samples by epifluorescence microscopy.

Sanger sequencing and nucleotide BLAST analysis confirmed that all seven *G. duodenalis* isolates from meats aligned with Assemblage B. Four of the confirmed sequences were from ground beef or chicken breasts, and three were from veal. NCBI accession numbers for *G. duodenalis* are PX349468 to PX349474.

Sequencing was only successful with two of the ten *Cryptosporidium* isolates, and BLAST analysis confirmed that both were *C. parvum*. One of the sequenced isolates was from ground beef and the other was from veal. NCBI accession numbers for *C. parvum* are PZ320970 and PZ320971.

## 4 Discussion

While many previous studies done worldwide have reported on the prevalence of *G. duodenalis* and *Cryptosporidium* spp. in a variety of foods, particularly fresh produce and bivalve shellfish, very few such studies have reported on the prevalence of these parasites in meats. In order to address this knowledge gap, and to support future risk assessments, the present study investigated the PCR prevalence of *G. duodenalis* and *Cryptosporidium* spp. in meats purchased at retail in Canada.

*Giardia duodenalis* and *Cryptosporidium* spp. DNA was detected by PCR and DNA sequencing in retail meat samples collected in three Canadian provinces. The number of positive samples was relatively low in all meat types, although ground beef was significantly more likely to be contaminated than the other meats tested, at least with *Cryptosporidium* spp.

As *G. duodenalis* and *Cryptosporidium* spp. typically inhabit the lumen and epithelial cells, respectively, of the small intestine in humans and animals, the detection of their DNA in a variety of retail meats was somewhat unexpected, and is likely indicative of cross-contamination from the environment. Meat animal carcasses, and subsequent cuts of meat, may become contaminated directly by fecal contact in the abattoir, most likely from the intestinal contents of the slaughtered animals, or possibly from infected human workers. It is also feasible that this contamination could occur through washing or processing the carcasses and meats with improperly treated water contaminated with cysts or oocysts. Robertson (2014) also suggested the possibility that meat could become contaminated by flies or other vectors.

However, the actual sources of contamination of the retail meats in the present study are not entirely clear. Molecular characterization of the isolates confirmed the presence of *G. duodenalis* Assemblage B and *C. parvum.* The presence of *G. duodenalis* Assemblage B may be more suggestive of direct or indirect contact with human feces as it is the predominant assemblage in humans, and is less commonly reported in farm animals (Dixon, 2021). Conversely, *C. parvum* is an important zoonotic parasite, and is very common in livestock, especially young animals (Ryan et al., 2021), suggesting that cross-contamination from feces to carcass may occur. Considering the high prevalence of *Cryptosporidium* spp. reported in cattle, the fact that *Cryptosporidium* spp. DNA was significantly more likely to be found in ground beef than in the other meats tested in the present study may support this particular source of contamination. However, the transfer of *Cryptosporidium* spp. oocysts from the feces of livestock to beef carcasses during slaughter was investigated by Moriarty et al. (2005). These authors reported that, while fecal bacteria from cattle can contaminate the rump and brisket area of carcasses during slaughter, they were unable to observe any transfer of *Cryptosporidium* spp. oocysts.

Together, *C. hominis* and *C. parvum* account for the majority of human infections worldwide. *Cryptosporidium hominis* has been reported in numerous animals, but most cases of human infection are thought to be transmitted anthroponotically (Ryan et al., 2021). While *C. parvum* can be transmitted to people both from animals and from other infected humans, with some subtypes (e.g., subtype IIc) almost exclusively transmitted anthroponotically, it is nevertheless considered to be the most important zoonotic species in humans, with calves, sheep and goats acting as major contributors to human infection (Ryan et al., 2021). While there are important regional differences in *Cryptosporidium* species predominance and sources of infection (Robertson et al., 2020), human *C. parvum* infections generally predominate in rural or agricultural regions where there is a greater likelihood for direct or indirect contact with livestock (Loeck et al., 2020; Ryan et al., 2021). As with *G. duodenalis*, the contamination of carcasses or meats with *C. parvum* from human feces may also occur, either directly through contact with infected workers at processing plants, butcheries or retail stores, or indirectly through the use of improperly treated water. Further genomic analyses to determine the *G. duodenalis* sub-assemblages and *C. parvum* sub-types present may be necessary to clarify the source, or sources, of contamination, but this was beyond the scope of this initial investigation.

An important limitation of the present study was that the prevalence of *G. duodenalis* and *Cryptosporidium* spp. in retail meats was based only on the detection of parasite DNA. Contamination of samples with DNA in the environment is always a consideration and must be controlled as much as possible. While the presence of *G. duodenalis* and *Cryptosporidium* spp. DNA is certainly indicative of contamination with cysts or oocysts, it does not confirm the presence of intact parasites, nor their viability or infectivity. We were, unfortunately, unable to confirm the presence of either *G. duodenalis* cysts nor *Cryptosporidium* spp. oocysts using epifluorescence microscopy performed on the concentrates of all sequence-confirmed PCR-positive samples. This may have been a result of the very small number of cysts and oocysts expected through cross-contamination. As the meat samples were frozen prior to testing, it is also possible that the cyst walls of *G. duodenalis* and the oocyst walls of *Cryptosporidium* spp. could have been fragmented through freeze-thawing. Since the commercial monoclonal antibodies used in this study target proteins on the cyst and oocyst walls, this fragmentation may have rendered them undiscernible. However, considering that the meats tested in this study are generally cooked before consumption, even the presence of viable *G. duodenalis* cysts or *Cryptosporidium* spp. oocysts would likely pose a minimal risk to consumers. Further study on the prevalence, genotypes and infectivity of enteric parasites in meats is warranted.

## CRediT authorship contribution statement

**Harriet Merks:** Methodology, Validation, Investigation, Data curation, Writing – review & editing, Supervision. **Jessica Remedios:** Investigation, Writing – review & editing. **Nicol Janecko:** Formal analysis, Resources, Writing – review & editing. **Brent R. Dixon:** Conceptualization, Resources, Writing – original draft, Supervision.

## Funding

No specific funding was received for this study.

## Declaration of competing interest

The authors declare that they have no known competing financial interests or personal relationships that could have appeared to influence the work reported in this paper.

## Data Availability

Data is contained within the article.

## Appendix A Supplementary data

Supplementary material: Supplementary Table 1. Primer sequences of the gene fragments amplified by PCR. Supplementary Table 2. Pairwise comparisons of *Cryptosporidium* spp. DNA in ground beef versus other meats tested
