# Supplementary material for: Giardia duodenalis and Cryptosporidium spp. in meat purchased at retail in Canada
Source: Food Waterborne Parasitol. 2026 Aug 26;44:e00357. doi: 10.1016/j.fawpar.2026.e00357 (PMC13551920; doi:10.1016/j.fawpar.2026.e00357)
Supplement: Supplementary file 1 [file mmc1.docx]

Supplementary Table 1. Primer sequences of the gene fragments amplified by PCR

| Gene (specificity) | Outer or nested PCR | Primer name | Primer sequence (5’-3’) | Reference |
| --- | --- | --- | --- | --- |
|  |  |  |  |  |
| 18S (*Giardia*) | outer PCR | Gia2029 | AAGTGTGGTGCAGACGGACTC | Applebee et al., 2003 |
|  |  | Gia2150c | CTGCTGCCGTCCTTGGATGT | Applebee et al., 2003 |
|  | nested PCR | RH11 | CATCCGGTCGATCCTGCC | Applebee et al., 2003 |
|  |  | RH4 | AGTCGAACCCTGATTCTCCGCCAGG | Applebee et al., 2003 |
| COWP (*Cryptosporidium*) | outer PCR | BCOWP F | ACCGCTTCTCAACAACCATCTTGTCCTC | Pedraza-Diaz et al., 2001 |
|  |  | BCOWP R | CGCACCTGTTCCCACTCAATGTAAACCC | Pedraza-Diaz et al., 2001 |
|  | nested PCR | COWP cry-15 F | GTAGATAATGGAAGAGATTGTG | Spano et al., 1997 |
|  |  | COWP cry-9 R | GGACTGAAATACAGGCATTATCTTG | Spano et al., 1997 |

Supplementary Table 2. Pairwise comparisons of *Cryptosporidium* spp. DNA in ground beef versus other meats tested

| **Pairwise comparison** | **OR** | **95% CI** | **p-value (Fisher's exact)** |
| --- | --- | --- | --- |
| ground beef vs. chicken breasts | 7.57 | 0.91 – 62.79 | 0.035 |
| ground beef vs. ground pork | 7.57 | 0.91 – 62.79 | 0.035 |
| ground beef vs. veal (assorted cuts) | 15.14 | 1.83 – 124.98 | 0.0022 |
| ground beef vs. all other meats combined | 10.09 | 2.56 – 39.83 | 0.0007 |
